# Towards diversity in science - a glance at gender disparity in the Brazilian Society of Neuroscience and Behavior (SBNeC)

**DOI:** 10.1590/1414-431X2020e11026

**Published:** 2021-07-16

**Authors:** F.S. Erthal, A.F. Bastos, C. Vaccariello, A.T.S. Madeira, T.S. Santos, J.B. Stariolo, L. Oliveira, M.G. Pereira, K.C. Calaza, C. Hedin-Pereira, E. Volchan

**Affiliations:** 1 Instituto de Biofísica Carlos Chagas Filho, Universidade Federal do Rio de Janeiro, Rio de Janeiro, RJ, Brasil; 2 Instituto Biomédico, Universidade Federal Fluminense, Niterói, RJ, Brasil; 3 Instituto de Biologia, Universidade Federal Fluminense, Niterói, RJ, Brasil; 4 Vice-Presidência de Pesquisa e Coleções Biológicas, Fundação Oswaldo Cruz, Rio de Janeiro, RJ, Brasil

**Keywords:** Gender disparity, Scientific meetings, Science societies, Neuroscience, Implicit bias, Stereotype threat

## Abstract

Gender equity is far from being achieved in most academic institutions worldwide. Women representation in scientific leadership faces multiple obstacles. Implicit bias and stereotype threat are considered important driving forces concerning gender disparities. Negative cultural stereotypes of weak scientific performance, unrelated to true capacity, are implicitly associated with women and other social groups, influencing, without awareness, attitudes and judgments towards them. Meetings of scientific societies are the forum in which members from all stages of scientific careers are brought together. Visibility in the scientific community stems partly from presenting research as a speaker. Here, we investigated gender disparities in the Brazilian Society of Neuroscience and Behavior (SBNeC). Across the 15 mandates (1978-2020), women occupied 30% of the directory board posts, and only twice was a woman president. We evaluated six meetings held between 2010 and 2019. During this period, the membership of women outnumbered that of men in all categories. A total of 57.50% of faculty members, representing the potential pool of speakers and chairs, were female. Compared to this expected value, female speakers across the six meetings were scarce in full conferences (χ^2^(5)=173.54, P<0.001) and low in symposia (χ^2^(5)=36.92, P<0.001). Additionally, women chaired fewer symposia (χ^2^(5)=47.83, P<0.001). Furthermore, men-chaired symposia had significantly fewer women speakers than women-chaired symposia (χ^2^(1)=56.44, P<0.001). The gender disparities observed here are similar to those in other scientific societies worldwide, urging them to lead actions to pursue gender balance and diversity. Diversity leads not only to fairness but also to higher-quality science.

## Introduction

The benefits of gender, ethnic, and cultural diversity go far beyond the academic milieu, harnessing the power of different views and backgrounds for innovation, discoveries, and profit. A representative workforce contributes to an inclusive science with the potential to pursue problems that are not even mentioned in mainstream academic discussions (1). As stated by Nielsen et al. (2), “...*Encouraging greater diversity is not only the right thing to do: it allows scientific organizations to derive an ‘innovation dividend’ that leads to smarter, more creative teams, hence opening the door to new discoveries”*.

In all fields of science, women are underrepresented in high academic ranks, and gender equity is far from being achieved in most academic institutions worldwide (3). In what is known as a “scissor graph”, in Brazil (see (4)), as in other countries (e.g., (5)), women slightly outnumber men as undergraduate and graduate students, but the balance inverts and the numbers drop dramatically for women relative to men in the higher and more prestigious stages of career and of academic status in the hierarchical scientific system.

These gender disparities are, to a large extent, associated with two important and interrelated factors: implicit bias and stereotype threat. Much of our mental processing works implicitly, or at least without conscious attentional focus. Implicit associations affect our decisions and behavior and are more predictive of attitudes than individuals' explicit beliefs (6). Implicit stereotypes are defined as the implicit associations of specific characteristics to members of a social group (defined by gender, race, age, ethnicity, appearance, and many other factors). They are derived from our life experiences and are shaped by the environment (including the social, cultural, and political environments; the media and news programs) (7).

When one is evaluating an individual's capacity, say his/her academic performance, and the individual belongs to a social group culturally associated with poor performance, this implicit stereotype has been shown to negatively bias the evaluation. Implicit stereotype is considered one of the most important driving forces concerning gender disparities in academic performance. Stereotypes of women do not fit the perceptions of the qualities of successful scientists (8). Attribution of lack of intelligence and lack of capacity for science is instilled early in life (9), spreads to educational and work environments, and remains stable over the years (10).

Evaluation of women's academic performance can be significantly undermined due to stereotypes, even outside the evaluator's awareness of such bias. This has been observed in experimental paradigms in which participants (faculty members) received a *curriculum* to be rated. The name on the *curriculum* was used to manipulate gender (man/woman) (11,12). Although the *curriculum* contents were identical, female names biased the evaluations towards significantly less competence. Faculty participants, both men and women, showed similar implicit negative biases against women (11).

Stereotype threat adds more obstacles to women's pursuit of an academic career. Spencer et al. (13) reviewed the insidious effects of stereotype threat and remarked that *“When members of a stigmatized group find themselves in a situation where negative stereotypes provide a possible framework for interpreting their behavior, the risk of being judged in light of those stereotypes can elicit a disruptive state that undermines performance and aspirations in that domain”*. Social evaluative tasks *per se* can evoke fear of exclusion - causing alarm, triggering a full blow of defensive reactions, and impairing performance. Stereotype threat escalates the fear of exclusion, further undermining performance (14,15). Contexts of social rejection or exclusion have been shown to activate the same brain regions as physical pain, leading neuroscientists to tie the word “pain” to both physical and social wounds (see review in (16)). These close links between neural systems processing physical pain and pain related to rejection, exclusion, and loss seem to be a plausible substrate for performance impairment under either physical or social pain. In fact, priming women with culturally based cues of stereotype threat negatively impacts their working memory capacity (17) and significantly blocks their performance in math tests (18) and in leadership tasks (19). Notably, stereotype threat can also spill over, resulting in a negative impact in other unrelated activities (20).

Combining the wide range of studies on implicit bias and stereotype threat and observations of the contexts in which performance measures are usually assessed in academia leads one to conclude there is a systematic underestimation of the true capacity and potential of people in groups that are negatively stereotyped in intellectual settings. These obstacles, to be overcome in academic career, unrelated to merit, certainly undermine aspirations in the stereotyped domain (13,21).

Authors have proposed that scientific societies may play an important role in pursuing gender equity (22,23) since some characteristics of these societies are favorable to leading this effort forward. To become a member of a society, one can apply individually without going through a selection committee, unlike positions (or fellowships) in research institutions. Additionally, societies board posts are usually voted for within an assembly of members (which includes students and early career members). Societies meetings are the forum in which members from all stages of scientific career are brought together and are a stimulus for student participation.

Academic meetings promote the building of professional networks and the sharing of new discoveries but are also an opportunity for early career women in academia to identify role models, form implicit images of a successful career in academia, and detect what is valuable in science (24).

An important step in putting a society in this leading position is to survey and divulge the gender imbalance in its own membership and boards. Also important is to engender a detailed scrutiny of its scientific meetings. Here, we study the Brazilian Society of Neuroscience and Behavior (SBNeC), the most representative association of neuroscientists in Brazil, with a special focus on its annual meetings. Neuroscience is a widely interdisciplinary field that addresses, among other themes, the processes that impact social interactions and the complex organization of human societies. This interdisciplinary field is increasingly contributing to reveal underpinnings and consequences of the underestimation and exclusion of negatively stereotyped groups, as well as helping to suggest proposals of best practices to foster diversity (6,7,14–17,20,25).

Recently, a paper by Corona-Sobrino et al. (26) proposed a more comprehensive model to assess gender gap in academic events, broadening the approaches to compile data on organizational structure and women's participation and attitudes. Here, due to limited data availability, we focus on gender balance among speakers at the society's meetings and the composition of the directory boards.

## Material and Methods

### SBNeC directory boards

The SBNeC has had 15 executive boards since its foundation in 1978 up to the 2017-2020 mandate. The board is composed of four posts: president, vice-president, secretary, and treasurer, elected at the annual meeting when previous mandates expired, with votes from all members present. From the society's website, we verified the composition of the directory boards by counting the number of men and women occupying each post during each mandate.

### SBNeC meetings

We examined each of six annual meetings of the SBNeC spanning from 2010-2019. We only included national and stand-alone meetings of the SBNeC, which were held in 2010, 2013, 2014, 2017, 2018, and 2019.

The meeting programs were downloaded from the SBNeC website. We recorded the gender of speakers at full conferences (the most prestigious lectures with only one speaker) and at symposia (in which a chair invites three/four speakers), as well as the gender of symposia chairs. Gender classification was based on the common usage of their first name. When unclear, we searched for pictures and pronouns in the *curricula* database of the National Brazilian Research Council (CNPq), in their respective institution websites, and in the Research Gate website.

We searched the SBNeC membership composition by gender in two years: 2010, which was the year of the first of the six meetings evaluated, and 2019, the year of the last meeting evaluated. The information was provided by the society's staff separately for faculty members at academic institutions, post-docs, graduate students, and undergraduate students (Table 1). Women outnumbered men in all membership categories. Faculty members at academic institutions, which we refer to as senior members, represented 57.50% (the average of 2010 and 2019) of the total membership. This is the expected proportion of women speakers and chairs.

**Table 1:** Membership composition by gender and category for 2010 and 2019 of the Brazilian Society of Neuroscience and Behavior (SBNeC).

	2010			2019		
	Women	Men	Total	Women	Men	Total
Undergraduate	103 (59.5%)	70 (40.5%)	173 (100%)	197 (68.2%)	92 (31.8%)	289 (100%)
Graduate (MSc+PhD)	203 (68.1%)	95 (31.9%)	298 (100%)	263 (69.9%)	113 (30.1%)	376 (100%)
Postdocs	17 (73.9%)	6 (26.1%)	23 (100%)	23 (79.3%)	6 (20.7%)	29 (100%)
Faculty members at academic institutions	44 (56.4%)	34 (43.6%)	78 (100%)	58 (58.6%)	41 (41.4%)	99 (100%)
Total	367 (64.2%)	205 (35.8%)	572 (100%)	541 (68.2%)	252 (31.8%)	793 (100%)

#### Speakers

For each of the six annual meetings, the percentage of female speakers was computed separately for full conferences and for symposia. Chi-square tests were performed to compare the observed percentage of female speakers in full conferences and symposia across the six meetings with the expected 57.50% of female senior members.

#### Chairs of symposia

The percentage of women chairing symposia was computed for each of the six meetings. A chi-square test was performed to compare the observed percentage of women chairing symposia across the six meetings with the expected 57.50% of female senior members.

#### Women-chaired vs men-chaired symposia

We investigated gender balance in symposia composition when either men or women were the chairs. Symposia coordinated by two chairs of different genders were excluded from the analysis. Those with more than one chair of the same gender were computed as one case. Here, we did not analyze individual meetings but summed the numbers of men and women speakers at symposia of all the six meetings. A 2×2 table contained the actual number of men and women in symposia chaired by women, and the actual number of men and women in symposia chaired by men. A chi-square test was performed.

All statistical analyses were performed with Statistica software (v.13, TIBCO Software Inc., USA), and a P*-*value less than 0.05 was considered significant.

## Results

### SBNeC directory boards

**Figure 1:**
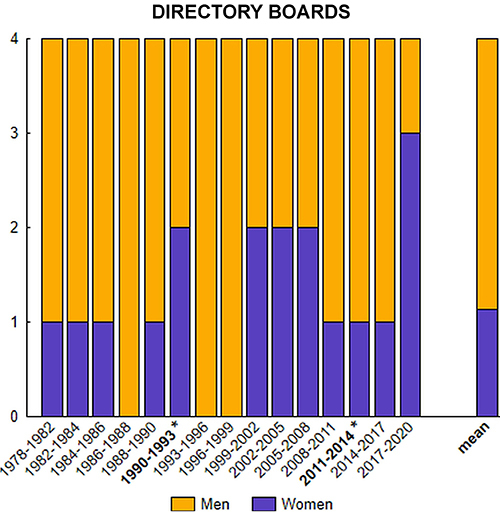
Directory boards of the Brazilian Society of Neuroscience and Behavior (SBNeC). The four posts (y-axis) are: president, vice-president, secretary, and treasurer. The asterisk indicates the two mandates in which a woman was the President of the Society.

Since its foundation, there have been 40 men and 20 women participating on the directory boards. The number of men and women in each mandate are depicted in Figure 1. The average count for the number of women filling posts on the directory board is 1.2 (30%), as depicted on the right side of Figure 1. Gender of members of the directory boards were unevenly distributed across the mandates, and the society has had only two women as president (mandates 1990-1993 and 2011-2014). Table 2 shows the total numbers and percentages of women and men in each post on the directory board (president, vice-president, secretary, treasurer) during the 15 mandates.

**Table 2:** Directory boards composition by gender and posts summing up the 15 mandates (1978 to 2020) of the Brazilian Society of Neuroscience and Behavior (SBNeC).

	Women	Men	Total
	N (%)	N (%)	N (%)
President	2 (13.3)	13 (86.7)	15 (100)
Vice-President	5 (33.3)	10 (66.7)	15 (100)
Secretary	6 (40.0)	9 (60.0)	15 (100)
Treasurer	5 (33.3)	10 (66.7)	15 (100)

### Speakers at meetings

At each annual meeting, the directory board indicates a scientific committee to evaluate the activities proposed by the members. Full conferences may be submitted by a member and/or by the directory board. Any member of the SBNeC can submit their own name for chair, the symposium’s theme, and the names of speakers to be invited.

**Figure 2:**
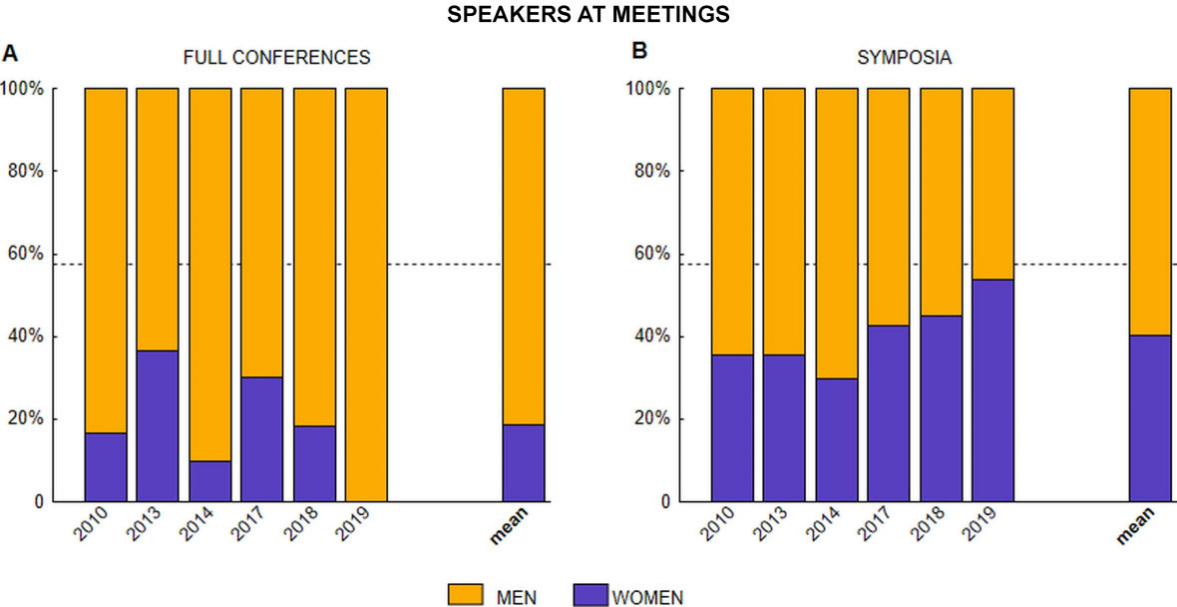
Speakers at full conferences (**A**) and at symposia (**B**) in six national and stand-alone meetings of the Brazilian Society of Neuroscience and Behavior (SBNeC). Dashed lines indicate the mean percentage of senior women members (57.50%) between 2010 and 2019.

For full conferences, the relative percentage of speakers by gender at the analyzed meetings is depicted in Figure 2A. Women speakers were scarce in all years (mean 18.54%), reaching a maximum (36.36%) in 2013 and falling to zero in 2019. Strikingly, this percentage is very far from the available percentage of senior women of 57.50%. The percentage of women speakers at full conferences across the six evaluated meetings (mean=18.54, SD=13.185) differed significantly from the 57.50% pool of senior women (χ^2^=173.54, P<0.001, df=5).

Gender balance for symposia speakers is shown in Figure 2B. In the first three analyzed meetings (2010, 2013, and 2014), the percentages of women speakers were below 36%. Women's participation from 2017 to 2019 suggests an increase towards 50%. Nevertheless, the percentage of women speakers in symposia across the six meetings (mean=40.35, SD=8.462) differed significantly from 57.50% (χ^2^=36.92, P<0.001, df=5).

### Chairs of symposia

**Figure 3:**
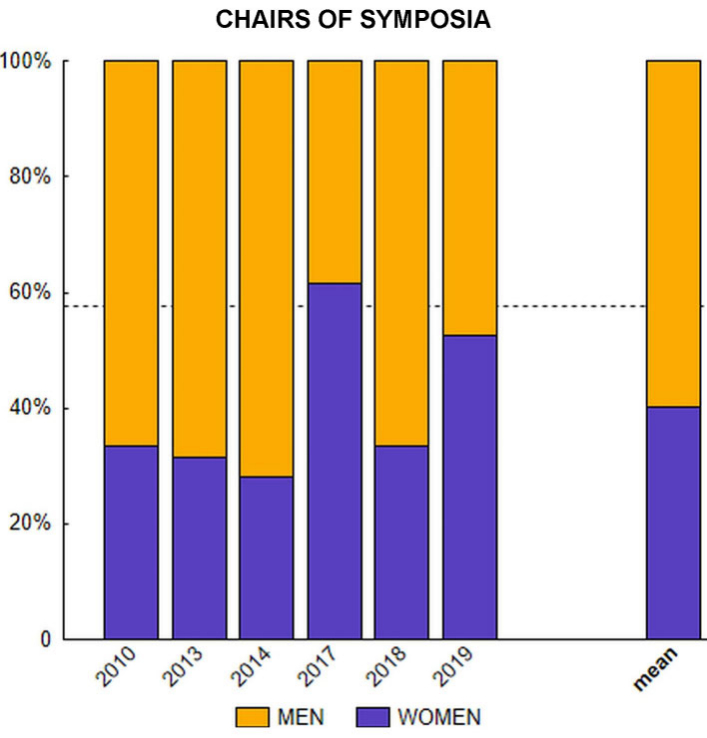
Chairs of symposia in six national and stand-alone meetings of the Brazilian Society of Neuroscience and Behavior (SBNeC). The dashed line indicates the mean percentage of senior women members (57.50%) between 2010 and 2019.

Gender balance for symposia chairs is presented in Figure 3. The percentage of women chairing symposia across the six meetings (mean=40.07, SD=13.619) differed significantly from 57.50% (χ^2^=47.83, P<0.001, df=5).

### Women-chaired *vs* men-chaired symposia

**Figure 4:**
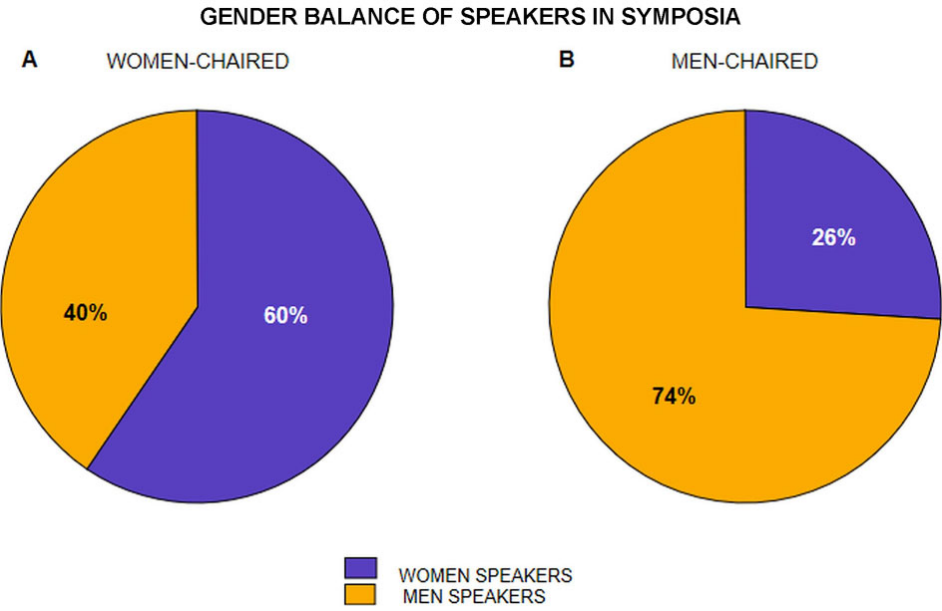
Gender balance of speakers in women-chaired symposia (**A**) and men-chaired symposia (**B**). Percentages were calculated by summing the number of male and female speakers at all six evaluated meetings

Summing the numbers of speakers at symposia of all the six meetings, at women-chaired symposia, there were 110 (59.46%) female speakers and 75 (40.54%) male speakers (Figure 4A). At men-chaired symposia, there were 87 (26.05%) female speakers and 247 (73.95%) male speakers (Figure 4B). Statistical analysis revealed that gender of chairs significantly influenced gender balance of speakers (χ^2^=56.44, P<0.001, df=1).

## Discussion

The present study considered one facet of diversity by examining gender representation in the SBNeC. We explored the composition of the directory boards since the society's foundation in 1978, as well as the gender ratio of speakers (full conferences and symposia) and of symposia chairs at the annual meetings from 2010 to 2019.

Women have been underrepresented in the directory boards across mandates, averaging 30% of the filled positions. Critically, the presidency post has been occupied by a woman only twice across the 15 mandates. These results are similar to those provided by a thorough investigation of gender balance in leadership posts of zoology societies worldwide (23). In the group of 202 societies addressed by these authors, the global estimation of women on directory boards was 25%. In their study, among other factors, societies with clear statements on gender equity, which are envisaged to help more women feel included in scientific leadership, were the ones showing the most gender-balanced boards (23). This is an important encouragement to pursue discussions and actions towards diversity within scientific societies.

SBNeC memberships, as scrutinized in 2010 and in 2019, revealed a predominance of women in both senior and early career positions. The surplus of women among senior members was not reflected in the gender balance of speakers during the annual meetings. Indeed, the percentage of female speakers was significantly different from the senior women's “availability”. Analyses of six meetings between 2010 and 2019 showed, on average, that less than 20% of speakers at full conferences were female. This percentage is extremely low considering that senior women comprised 57.50% of the membership pool.

Across symposia, the discrepancy was still present, with the percentage of female speakers significantly below the percentage of women in the seniors' pool. Considering that post-docs also participate as speakers in symposia and women accounted for 73.9% of this category in 2010 and 79.3% in 2019 (see Table 1), the pool of available invitees, and consequently the gender discrepancy among speakers, is enlarged even further.

Gender bias is a concern in the field of neuroscience (27,28) as well as in other areas of research. Consistent with the data presented here, women remain highly underrepresented in the most prestigious presentations in meetings of many scientific societies (29,30), even when, as in SBNeC, female members predominate (31).

As mentioned before, gender-based stereotype threat is even more pervasive in academic environments in which men typically predominate in prestigious positions. Men's prevalence in societies' boards and as speakers at societies' meetings, absence of childcare facilities, and additional costs when caregivers are needed create an even more hostile environment for women's engagement in higher status roles (22,26,32). This is an important issue inasmuch as recent studies have shown that the COVID-19 pandemic will increase the gender gap in science, especially for mothers of young children (33). These obstacles act to undermine the motivation to participate as speakers, which unfortunately decreases women's visibility. Further, scientific evidence for negative implicit bias when judging women's performance (11,12) reinforces gender-based stereotypes in science and makes it more difficult for women to become visible.

The prevalence of men as symposia chairs found in the current study is similar to findings for meetings of other societies (29,31). This presents an additional problem since, in the SBNeC and other societies, symposia chaired by men have a significantly lower proportion of women speakers than in women-chaired symposia (29,31). Implicit perceptions of women's “inferiority” in science and the stronger association of men with scientific careers (8) may generate an implicit bias for male chairs to avoid women and invite those similar to themselves as speakers. Although the negative implicit bias against women's capacity for science also operates among women who evaluate women (11); data by Handley et al. (34) showed that women are more prone to rely on scientific evidence of gender biases in science. Therefore, we hypothesize that women are becoming more conscious of the importance of having a more expressive representation. Once women overcome obstacles and decide to chair a symposium, their selection of speakers is likely to pull more women out of invisibility (35).

Exposure to role models is effective as a form of counter-stereotyping both in the short and long run (36). Increasing the presence of women presenters at full conferences and in leadership positions can encourage other women to follow (37). This could help fight the implicit bias that being a woman and being in leadership (or being academically successful) are mutually exclusive. Indeed, efforts directed towards expanding the number of women and other underrepresented groups have become a major concern for many scientific societies (22,23,29–31,38) as well as editorial boards for the most prestigious scientific journals worldwide (1,39,40).

### Limitations

Absence of data precluded attempts to include other stereotyped groups. We had to adopt a binary gender classification, and further important ethnic/racial considerations could not be taken into account.

A wider picture of gender bias in SBNeC meetings was precluded by the absence of data. Proposals accepted/refused by the scientific committees and their criteria and decision processes were unavailable, as well as accepted/refused invitations from chairs.

### Conclusions

Achieving gender equity is still a great challenge. Scientific societies are in a good position to lead the needed changes in academia. During the last decade, efforts have been made to foster the discussion of this topic in SBNeC meetings. These debates helped to raise awareness of the urgency of achieving gender balance and culminated in the establishment of a Committee on Diversity in 2020 and the election of a woman as the president of the society for the 2021-2024 mandate.

Negative implicit bias and stereotype threat have been important factors that detain or undermine the academic representation of stereotyped groups. Collecting data and divulging these imbalances is within the realm of contributions to change the present status. We hope with the present work to stimulate even more societies to prioritize this discussion and to work towards a more diverse community in science.

#### Recommendations and future directions

To move forward with the purpose of achieving gender equity of speakers at scientific meetings, we support the recommendations of Martin (32), which are summarized in the ten simple rules reproduced below:

“...*(i) Collect the Data; …(ii) Develop a Speaker Policy; ...(iii) Make the Policy Visible; ...(iv) Establish a Balanced and Informed Program Committee; ...(v) Report the Data; ...(vi) Build and Use Databases; ...(vii) Respond to Resistance; ...(viii) Support Women at Meetings; ...(ix) Be Family-Friendly; ...(x) Take the Pledge*” (32).

Furthermore, the SBNeC should make available guidelines for increasing diversity in a broad sense on its website, reinforcing its decision to embrace inclusion and diversity. The Committee on Diversity should be strengthened and work towards awareness of implicit bias by divulging the present data and related works. SBNeC should encourage its members to complete the Implicit Association Test (7) to recognize their own implicit bias. This can help to avoid implicit bias, for example, in their invitations to speakers and when proposing activities for meetings.
